# Audio-Based System for Automatic Measurement of Jump Height in Sports Science

**DOI:** 10.3390/s19112543

**Published:** 2019-06-04

**Authors:** Basilio Pueo, Jose J. Lopez, Jose M. Jimenez-Olmedo

**Affiliations:** 1 University Institute for Computing Research, University of Alicante, 03690 Alicante, Spain; 2 ITEAM Institute, Universitat Politecnica de Valencia, 46022 Valencia, Spain; jjlopez@dcom.upv.es; 3 Physical Education and Sport, University of Alicante, 03690 Alicante, Spain; j.olmedo@ua.es

**Keywords:** jump, measurement, audio, sports, validation, instrument, algorithm

## Abstract

Jump height tests are employed to measure the lower-limb muscle power of athletic and non-athletic populations. The most popular instruments for this purpose are jump mats and, more recently, smartphone apps, which compute jump height through manual annotation of video recordings to extract flight time. This study developed a non-invasive instrument that automatically extracts take-off and landing events from audio recordings of jump executions. An audio signal processing algorithm, specifically developed for this purpose, accurately detects and discriminates the landing and take-off events in real time and computes jump height accordingly. Its temporal resolution theoretically outperforms that of flight-time-based mats (typically 1000 Hz) and high-speed video rates from smartphones (typically 240 fps). A validation study was carried out by comparing 215 jump heights from 43 active athletes, measured simultaneously with the audio-based system and with of a validated, commercial jump mat. The audio-based system produced nearly identical jump heights than the criterion with low and proportional systematic bias and random errors. The developed audio-based system is a trustworthy instrument for accurately measuring jump height that can be readily automated as an app to facilitate its use both in laboratories and in the field.

## 1. Introduction

Jump height is a commonly used measure of lower-limb muscle power [1] and coordination of lower and upper extremities [2]. For athletic and non-athletic individuals, sports professionals are able to study neuromuscular and performance qualities by monitoring changes in jump height, which correspond to variations in functional performance [3].

The number and typology of instruments to assess lower body power through vertical jump tests is considerable and can be categorized into three methods. First, jump height can be obtained by means of numerical integration of ground reaction forces measured with force plates [4]. Second, the center of gravity of the body can be tracked by biomechanical motion capture to follow excursion in jump executions [5,6]. Finally, the time span between take-off and landing can be transformed into jump height by means of basic linear kinematics with timekeeping instruments that measure flight times of athletes [7]. According to this equation, since jump height is proportional to the square of flight time, the precision of jump height is related to an accurate timekeeping measurement.

The latter instruments are very popular among sports professionals owing to its ease of use, portability and low cost compared to laboratory equipment such as force plates and motion capture systems. Flight time assessment from which to calculate the displacement of center of gravity during flight (jump height) requires instruments with an accurate selection of take-off and landing time events [8]. Jump mats, also known as contact mats, were the first instruments of this kind, consisting on a mat that operate as an electric switch, pressure-activated by athlete weight [9]. Alternatively, photocell mats were introduced as a bar with an array of emitting infrared (IR) diodes aligned with a secondary bar with receiving IR diodes to create an optical barrier that athletes interrupt when executing jumps [10,11]. More recently, smartphone apps using high speed video recordings of athlete’s feet have allowed users to manually select take-off and landing frames from which to compute flight time by counting the number of frames between such time events [12]. These apps have become widespread in sports sciences due to the intrinsic advantages of smartphones, such as portability, connectivity, and data processing.

However, while estimation of jump height through flight time is a validated procedure [9], there are a number of drawbacks in manual digitization of events by video observation. The sampling frequency of the image sensor in smartphones (video frame per second, fps) is low for the temporal resolution required to select the key frames defining flight time. Moreover, take-off and landing phases of a vertical jump show the maximum velocity values of jump execution [13], so typical values of 120 or 240 fps of slow-motion videos in the latest models of iPhone result in undersampling. Further, shutter speeds are usually not accessible to operate by the user, and the fastest shutter speed, which is around 1/1000 s in advanced smartphone models, is set automatically only in brightly lit scenes, such as in sunlight. Consequently, most of the jump trials, which are performed indoors or in poorly lit environments, will result in slightly blurred images of feet in take-off and landing phases [14]. For example, with a jump height of 40 cm, the take-off velocity is 2.8 m/s [13], which causes a motion blur of about 1.1 cm in highest speed video of 240 fps when shutter is 1/240 for maximum light. This motion blur will smear across 28 pixels vertically in a typical frontal image of a foot 30 cm high, recorded at 720p resolution. All these issues play a role in the accuracy of measurements, since human observation is paired with hesitation in selecting the right frame. For instance, for the jump execution of the previous example, an observation inaccuracy of only one frame for both take-off and landing introduces an error of 1.2 cm. Still, if observers were provided with images without technical flaws, there would arise observation mismatches between different observers. For example, measurements with two observers may show different errors, as one can make more precise assessments than the other but may also differ systematically due to bias between observers, or the so-called observer effect [15]. Moreover, the outcome of such systems is achieved after some human intervention to analyze jumps manually, so the process lacks the necessary speed for typical sessions comprising a large number of athletes being tested. Therefore, apps based on manual digitization by video observation have several drawbacks to being considered valid alternatives to other instruments, such as jump mats.

To overcome the technical and observation problems associated with the manual digitization of video recordings, this study developed a non-invasive instrument that automatically extracts take-off and landing instants from an audio recording of jump execution. Such audio recordings can be carried out via the built-in microphones of smartphones. The developed algorithm analyzes the sound pattern to detect take-off and landing instants in real time by means of signal processing and computes flight time accordingly. The typical audio sampling frequency of 48,000 Hz used in current smartphones provides this instrument with high time resolution. Additionally, the unattended extraction of flight time aims at removing errors due to human observation. The applicability of the instrument was tested by comparing the measured jump heights with those of a commercial jump mat system.

## 2. Materials and Methods

### 2.1. Experimental Procedure

Fourty-three active athletes in various disciplines participated in the present study (mean ± SD: age 24.1 ± 2.6 years, body weight 75.1 ± 8.7 kg, body height 177.2 ± 6.8 cm). Subjects were told not to drink alcohol or caffeinated beverages during 24 h before the test. None of them showed any lower-limb injury nor were they medicated. The study protocol conformed to the guidelines of ethical principles of the Declaration of Helsinki and was approved by the Human Research Ethics Committee of the University of Alicante (IRB No. UA-2019-02-25). Each subject signed a written informed consent before participation. The study consisted of repeated measurements of jump height on subjects during a single test session. A 5 min standardized warm-up session on a cycle ergometer (Cardgirus Pro Medical, Alava, Spain) at 80 W power and 60 to 65 rpm cadence was performed prior to jump executions. Subjects were then instructed with familiarization trials to attain a correct jumping technique. Next, subjects performed 5 countermovement jumps repetitions [3] with a 1 min rest period between trials. All executions were monitored for proper technique, so only successful trials were considered. Subjects were told to perform jumps at different heights to collect a wide range of jump height measures.

**Figure 1:**
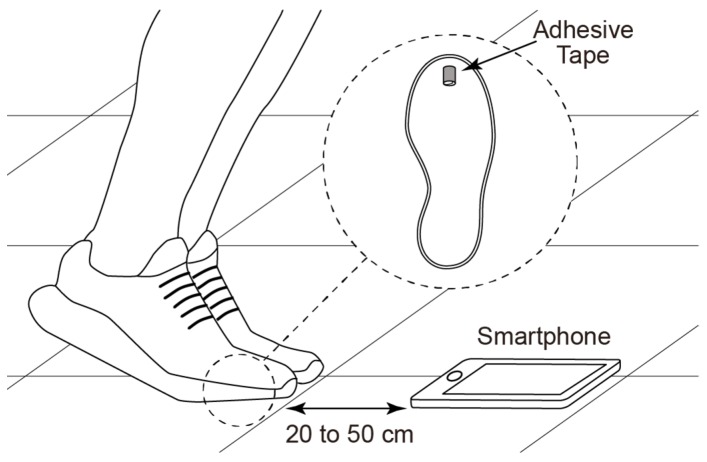
Experimental setup of jump recordings with the microphone of a smartphone.

In this study, an audio capture system was used to record the sound wave generated by an athlete when executing a vertical jump. The aim was to automatically extract take-off and landing phases, so the microphone was set to capture the audio waveform of both feet at these instants. For the take-off phase to be identified, a 5 cm piece of regular adhesive tape, usually made from rubber-based materials, is cut and stuck on the ends, leaving the adhesive side on the outside. This loop tape, about 2 cm long, is stuck to the sole of the shoe in the area of the ball of the foot, approximately at the end of the metatarsal bone and to the floor simultaneously to create a unique sound pattern. Athletes were required to use their own shoes to include in the experiment the variety of soles usually worn while training and to use a new piece of adhesive tape for each execution. The resistance created by the adhesive tape is negligible compared to force impaired at take-off. The size of the adhesive tape was roughly calculated by each user to check that this operation was not critical for the algorithm. The amount of error on flight time caused by slight changes in the adhesive tape size would be negligible given the feet’s take-off velocity. Figure 1 shows the experimental setup consisting of a smartphone recording of the sounds during take-off and landing and the position of the adhesive tape in the shoe sole. The smartphone is placed at the minimum distance to create a safe zone in order to prevent athletes from landing on the smartphone due to inadequate jump execution. In practice, typical distances from 20 to 50 cm are adequate to generate the sound pattern for the algorithm to work.

The landing phase is identified by the sudden sound pressure peak created as feet touch the ground. The requirement for a proper jump execution is to land with straight legs and both feet simultaneously [3]. In the case of landing with one foot slightly delayed with respect to the other, the algorithm to be developed should trigger the landing event when the first foot touches the ground, as this is same criterion used in force plates, jump mats, and observational instruments [12]. All measurements were performed with the audio system of a smartphone to simulate similar conditions to those of manual video digitization in smartphone apps. Three different smartphones were used in order to avoid any overfitting effect in the signal processing algorithm to the microphone and audio electronics of the smartphone. The sampling frequency was set to 48,000 Hz and the raw audio signals were transferred to a computer (Core i7, Windows 10) for analysis. The signals were recorded in different scenarios trying to reproduce the typical conditions of real sport-related facilities: workout rooms, gymnasiums, etc. In addition to this, corridors and offices were also tested as recording venues for a comprehensive analysis. Table 1 shows that some recording scenarios had low reverberation and moderate background noise, such as small fitness rooms, but other locations, such as gymnasiums, typically had long reverberation times and generally more background noise. In all scenarios, the signal-to-noise ratio of the recorded signal was very high due to the proximity of the smartphone to the athlete’s feet, where the audio signal is generated. Accurate noise levels and signal-to-noise ratios of the recording scenarios will be calculated in Section 2.3, once the algorithm has been described.

In order to test the accuracy of outcomes, all jump executions were simultaneously measured with a validated jump mat system (Globus Ergo Tester, Codogne, Italy) as a reference, which has been demonstrated to be a valid instrument for monitoring changes in jump performance [9].

### 2.2. Audio Signal Processing

Audio event classification and detection (AEC/D) has been an active field of research in recent years [16,17]. Both classification and detection is used in this work, the first study to distinguish take-off from landing and the second to precisely detect, in time, the occurrence of both events. Even though it has been accepted that audio event classification is easier to deal with than detection, recent works emphasize the importance of detection [18], which is of particular importance in this work. In fact, as discussed later, distinguishing take-off from landing will be straightforward, so more effort will be put on precise time detection. Besides, reverberation and background noise in typical sport-related scenarios can be major drawbacks. In addition to this, the algorithm must be able to process and give jump height automatically in real time. Therefore, the characteristics of the algorithm to be developed should be:

- Immunity to background noise in typical scenarios of use
- Not affected by the reverberation time of typical scenarios of use
- Moderate complexity to be used in real time on smartphones
- Totally unsupervised

**Figure 2:**
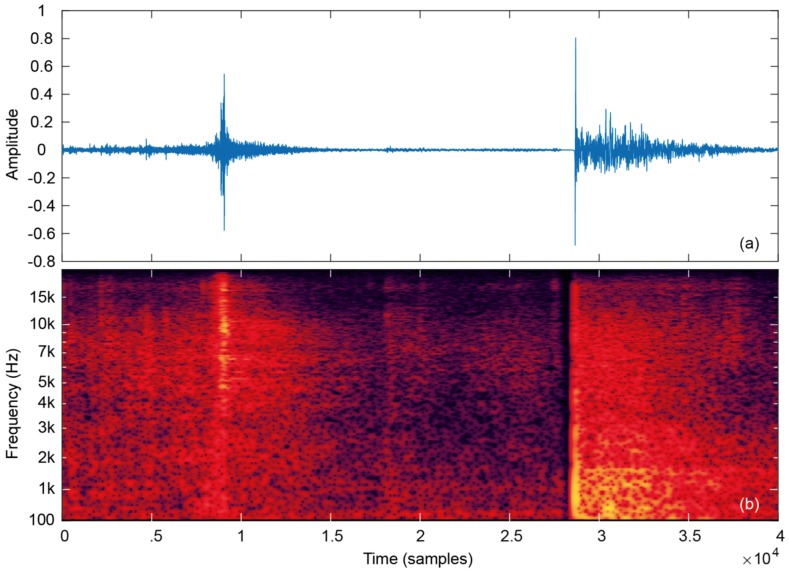
Typical jump audio signal showing take-off and landing events. (**a**) Waveform; (**b**) spectrogram.

The training signals were recorded in different scenarios to test the algorithm against various reverberation and background noise levels. In order to calculate flight time, the algorithm must be able to detect take-off and landing signals with precision and subtract both times. A preliminary analysis of the recorded jump signals both in time and frequency was carried out. Figure 2 shows the recorded sound of a typical jump execution with take-off and landing events, together with and the corresponding spectrogram. The rest of the collected jump signals were also inspected to ensure that the recorded sound followed a similar wave pattern.

In the time domain, the take-off signal, corresponding to peeling the adhesive tape, is a noisy burst than increases and decreases progressively with a Gaussian-like envelope. However, the landing signal is an impulsive signal that starts and decreases suddenly in time with a large amplitude value. The huge slew-rate at the beginning of the landing signal is present in typical surfaces of sport facilities, so this factor is not critical for a proper detection. In the frequency domain, the landing signal concentrates its energy from DC to about 4–5 kHz, whereas the take-off signal extends mainly from 3 kHz to 20 kHz. This different frequency composition will be exploited latter.

Since the sound level at landing, corresponding to the feet impact over the floor, is considerably larger than that at take-off, it seems reasonable to use landing as a triggering event to identify a jump execution. In this way, once the time position of the landing has been estimated, the algorithm will proceed to look for the take-off in a previous position. As it will show later, this task is more difficult due to the non-impulsive nature of the sound. According to the frequency composition of the sound at landing, a low-pass filter version of the signal can be used in order to distinguish this event from the take-off sound and also to suppress background noise at high frequencies. The detection of a landing event, which is very energetic, can be carried out by just integrating the energy during a certain period of time using a moving average and applying a threshold to this value over time. With the low-pass filtered version of the sound signal $x_{l}$, the average energy is defined as:

(1) $$e_{l}\left[n\right]=x_{l}^{2}\left[n\right]*w\left[n\right],$$

where w is a rectangular function of size *N* samples, rect*^N^*[*n*] = Π*^N^*[*n*], defined as:

(2) $$rect^{N}\left[n\right]=\Pi^{N}\left[n\right]=\{\begin{array}{ll} 0, & n<0\\ \frac{1}{N}, & 0\leq n<N\\ 0, & n\geq N \end{array}$$

On convolving the instantaneous energy of the signal defined by $x_{l}^{2}\left[n\right]$ by the rectangular function, a moving average effect is achieved with the desired resolution. With the landing event already detected, the beginning of the impulsive signal can be determined with great precision by directly inspecting the time signal to locate a big step point.

Once the landing point has been located, the take-off point must be searched in a prior time zone, which includes possible realistic jump signal patterns. According to the frequency composition of the take-off sound, a high-pass filter version of the signal can be used in order to distinguish this event from interference sounds in the venue and also to suppress background noise at low frequencies. The detection of the take-off event, consisting of a noisy burst, can be carried out by integrating the energy during a certain period of time using a moving average, and looking for the maximum point inside the aforementioned search zone before landing. Let $x_{h}$ be the high-pass filtered version of the sound signal, so the average energy is defined as:

(3) $$e_{h}\left[n\right]=x_{h}^{2}\left[n\right]*v\left[n\right],$$

where v is a rectangular function of size *M* samples, as previously defined.

**Figure 3:**
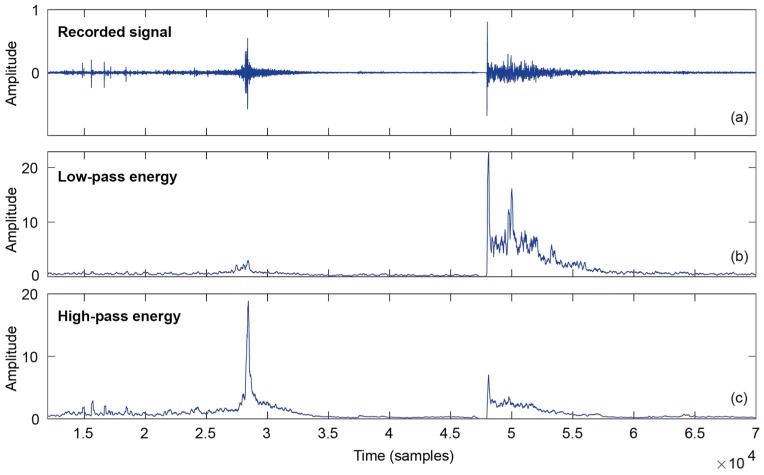
The recorded audio signal of a jump execution and filtered versions to enhance take-off and landing events. (**a**) Waveform; (**b**) low-pass energy; (**c**) high-pass energy.

Figure 3 shows the sound signal of the jump (a), and the resulting energy computation for low (b) and high frequencies (c). For this example, the low and high-pass filters are second-order Butterworth low-pass filter with the same cut-off frequency of 3 kHz. The small group delay variations with frequency produced by second order Butterworth filters, around one sample, produce an almost negligible effect on the accuracy of the algorithm. Nevertheless, the IIR (Infinite Impulse Response) filters can be replaced by linear phase FIR (Finite Impulse Response) filters for a very slight improvement, if computer power is available in the device. All the operations are carried out in 32 bit floating point to guarantee proper filter stability and a sufficient round-off for the calculation of energies. The moving average window lengths have been fixed for the first experiments at 3 ms for both w and v, equivalent to *N* = *M* = 144 samples. Figure 3b shows that $e_{l}$ can be a good landing indicator with enough amplitude difference from noise floor and take-off levels. Similarly, Figure 3c displays a large peak at the take-off position.

### 2.3. Noise and Signal Levels of Recording Scenarios

In order to validate the proposed algorithm against the background noises in typical scenarios of use, the mean values of noise, take-off, and landing signal for all recording scenarios are shown in Table 2. All recordings were performed with similar microphone sensitivity across smartphones and their values were not changed during recordings. Furthermore, any automatic gain control (AGC) was activated. Besides, jump signal levels were high and similar in all scenarios, since jumps were executed at similar distances from the smartphone.

Table 2 shows two signal-to-noise ratios, one for the low-pass filtered signal used in landing detection and another for the high-pass filtered signal used in take-off detection. For landing, the entire recorded signal is considered noise, with the exception of the sound of the landing event. For take-off, since only the possible jump window (0.2 to 0.8 s) before landing is explored, the recorded signal in this window is considered noise, except for the sound of the take-off signal. In both cases, the power of the signal is calculated by integrating an interval equivalent to the 3 ms smoothing window at the maximum amplitude of the event.

According to the results of Table 2, the three more complex and noisier scenarios (gymnasium, workout room, and noisy corridor) show very similar low frequency (up to 3 kHz) background noise of around −28 dB_FS_. In the first two scenarios, noises come from human activity and sports equipment, whereas in the noisy corridor, the hum of the computer fans was the main source of noise. Lastly, in the small fitness room, the background noise was very low, around −42 dB_FS_, since this type of room is typically low reverberant without major noise sources. As expected, the landing signal shows very similar levels (from −8.3 to −11.2 dB_FS_), with a difference of less than 3 dB between scenarios. This difference may be due to the different type of floor, or the contribution of some very early reflection, which indicates that the floor type is not a parameter that can significantly influence the algorithm. Besides, the signal-to-noise ratio for the first three rooms is about 18 dB, which provides a large margin to set the landing detection threshold and, therefore, is not a critical parameter for the proper functioning of the algorithm.

For the detection of take-off events, the high frequency background noise shows a much lower value than that of the low frequency noise, and the differences between rooms are negligible. The take-off signal level is lower than the landing signal, but since the noise in the high frequency band is also lower, the signal-to-noise ratio in the search area is even better than that in the landing area, around 23 dB.

Finally, it should be mentioned that, despite these wide margins, in the event of an extremely loud noise produced in the room, the algorithm could detect that noise like a jump. For this to happen, a loud noise with high low frequency content should be produced after a loud noise with high frequency content inside a window of 0.2 to 0.8 s. In the 215 jumps recorded in the typical scenarios, that circumstance never occurred, but in the very unlikely event that it occurs, the user simply discards the measurement and repeats the jump.

### 2.4. Optimization of the Algorithm Parameters

The basic characteristics of the algorithm have been already established. However, a proper validation requires us to test the algorithm with a number of jump execution signals and to optimize precision by adjusting its parameters. For this purpose, sound measurements have been carried out while simultaneously using a commercial jump mat system, as described in Section 2.1. To adjust the algorithm, 75 of the total 215 measurements taken (approximately one third) were randomly chosen.

As discussed earlier, the detection of the landing position is very precise, since it is a very clear impulsive signal. On the contrary, the detection of the take-off point is not so obvious, since it is a noisier signal spread over some milliseconds. Therefore, efforts in parameter optimization should be focused on the detection of the take-off signal. Two parameters can be modified at this stage, the length of the moving average and the cut-off frequency of the high-pass filter. Using the randomly-chosen 75 jump signals and the paired outcomes of the mat system, we have computed the difference between the flight time estimation provided by our algorithm and the jump mat lecture.

**Figure 4:**
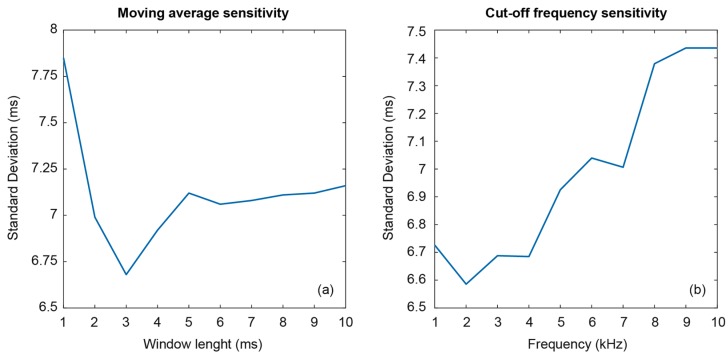
Algorithm sensitivity as a function of (**a**) moving average length and (**b**) cut-off frequency.

The first sensitivity test aimed at determining the optimum moving average length. Since the take-off signal lasts about 10 ms, a lower value has been chosen as a starting point. Ten window lengths from 1 to 10 ms have been verified, with a constant cut-off frequency of 3 kHz. Differences and standard deviations between estimation and jump mat lecture have been computed for the 75 jumps. Figure 4a shows the results, where it can be verified that the best value for the window length is 3 ms. Nonetheless, windows with longer lengths does not rise the error significatively but increase computational power, so 3 ms is the best option.

The second sensitivity test is intended for selecting a more appropriate cut-off frequency. Considering a moving average window of 3 ms, cut-off frequencies from 1 to 10 kHz have been tested in steps of 1 kHz. Figure 4b shows that the best value for the cut-off frequency is 2 kHz. However, both 3 and 4 kHz can also be considered to be optimum cut-off frequencies, since differences with respect to 2 kHz are negligible. Indeed, the signal produced on peeling the tape has low energy between 2 and 4 kHz, as can be seen in Figure 2b. Therefore, despite our experiments, 2 kHz produces minimum error and could be advantageous to increase cut-off frequency up to 3 or 4 kHz to improve noise immunity of the algorithm in very noisy scenarios. Accordingly, this parameter optimization achieves a slight error improvement, though not critical.

### 2.5. Final Algorithm for Flight Time Extraction

From the considerations of the previous points, the proposed algorithm for flight time estimation performs the analysis according to the following procedure:

1. The incoming signal is divided into two paths. In one path, a second-order Butterworth low-pass filter is applied with cut-off frequency of 3 kHz. In the other path, a second-order Butterworth high-pass filter is applied with a cut-off frequency of 3 kHz.
2. The energy of the signal is computed in each path and averaged in time with a moving average of 3 ms, equivalent to 144 samples at *f_s_* = 48 kHz.
3. The landing event is detected in the low frequency path by a fixed threshold. Once detected, a fine-tuning process is used to accurately detect the beginning of the impulsive signal looking for a big step in the time domain signal.
4. For finding the take-off event, a search zone prior to the landing event is defined. This zone includes possible realistic jumps from the smaller to the largest values and has been fixed between 0.2 to 0.8 s.
5. The maximum average energy in the high-frequency path inside the search area is considered the take-off event.
6. Finally, flight time is computed as the difference between the two events.

**Figure 5:**
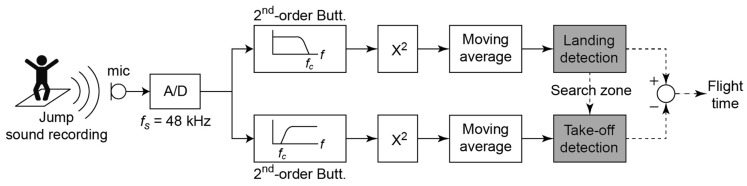
Block diagram of the signal processing algorithm. Solid arrows represent signal paths, whereas dashed lines represent information. A/D: Analog-to-digital converter.

Figure 5 shows the block diagram of the signal processing algorithm. The microphone of the smartphone captures the sound signal of the jump, which is converted to digital at a 48 kHz sample rate, passing through the different stages described earlier.

A piece of software was developed for this study (Matlab R2018) which provides waveform identification of jump execution, selection of take-off and landing events, extraction of time events and computation of flight time.

### 2.6. Instrument Validation

The characteristics of the collected jumps were reported with descriptive statistics (mean ± SD). To test the reliability of the proposed instrument in comparison with the jump mat system, a 2-way random single measurements (consistency and absolute agreement) intra-class correlation coefficient (ICC) (2,1) and Cronbach’s α was used [19]. The criteria to interpret ICC were as follows: poor (<0.5), moderate (0.5–0.75), good (0.75–0.9), and excellent (>0.9) reliability [20]. Additionally, paired sample *t*-tests and mean differences with a 95% confidence interval were computed to compare outcomes between the proposed audio-based system and jump mat, whereas the 95% confidence intervals represent uncertainty in the true value, and the smallest worthwhile change (SWC) depicts the minimum improvement likely to have practical impact. The criterion for SWC has been set to 0.2 of the between-subjects standard deviation [21]. The usefulness of the audio-based system was then assessed by comparing SWC and the typical error of measurement [22]. The agreement between the two instruments was also studied with Bland–Altman plots [23] through differences of outcome pairs against their mean values, to identify any random error, and proportional bias between instruments with bivariate Pearson’s product moment correlation coefficient as *r*^2^ > 0.1 [24]. Finally, to examine the validity of the instrument, bivariate Pearson’s product moment correlation coefficient (*r*) with 95% confidence intervals (CI) was used between audio-based and jump mat paired samples. The strength of the *r* coefficient was trivial (<0.1), small (0.1–0.3), moderate (0.3–0.5), high (0.5–0.7), very high (0.7–0.9), and practically perfect (>0.9) [25]. The standard error of estimate (SEE) was computed in raw units and standardized, evaluated via *r* to allow estimation of confidence limits [26]. The standardized SEE is interpreted using half the thresholds of the modified Cohen’s scale: trivial (>0.1), small (0.1–0.3), moderate (0.3–0.6), large (0.6–1.0), very large (1.0–2.0), and extremely large (>2.0) [25]. All statistical analyses were computed with an available spreadsheet for validity [27] and with SPSS v. 22 (IBM Corp, Armonk, NY, USA).

## 3. Results

In order to examine the agreement between the proposed system and a typical instrument used by sports professionals, a total of 215 jumps were collected from 43 participants performing 5 countermovement jump repetitions each. Descriptive statistics show the following mean flight times (mean ± SD): 451.9 ± 85.8 ms for the audio-based system and 453.7 ± 85.3 ms for the jump mat. Following a simple kinematic equation [3], *h* = *t*^2^*g*/8, jump height *h* can be calculated with flight time *t* and gravity acceleration *g* (9.81 m/s^2^). Computed jump heights from collected flight times resulted in mean values of 25.9 ± 9.5 cm for the audio-based system and 26.1 ± 9.5 cm for the jump mat.

The intra-class correlation coefficient between the two instruments show almost perfect agreement, both testing consistency and absolute agreement for flight time and jump height (ICC = 0.995–0.997), as shown in Table 3. Similarly, Cronbach’s α resulted in coefficients near unity, which shows excellent reliability. Flight time and jump height mean differences between instruments were 1.7 ms and 0.18 cm (*p* < 0.01), respectively.

In order to put into context such results, the smallest practical change in flight time or jump height, also known as SWC, is calculated as 20% of between-subjects SD (standardization), resulting in 17.1 ms and 1.9 cm, respectively. The uncertainty in measurements is given by the typical or standard error of estimate SEE: 7.2 ms and 0.81 cm for flight time and jump height, respectively. The standardized versions of 0.09 and 0.08 allow for interpretation as trivial. The relation between SWC and SEE results in 2.34 and 2.36 as the signal to noise ratio of practical measurement.

**Figure 6:**
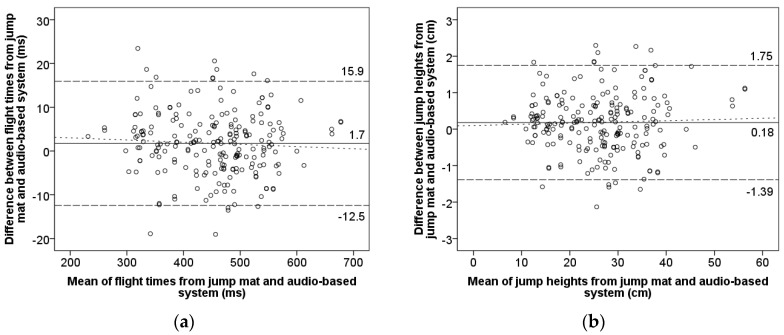
Bland–Altman plots for the measurements of the jump mat system and audio-based system. The solid central line represents mean between instruments (systematic bias); upper and lower dashed lines show a mean ± 1.96 SD (random error); the dotted line shows regression (proportional bias). (**a**) Flight time: regression *y* = −0.005x + 4 ms, *r*^2^ = 0.003; (**b**) Jump height: regression *y* = 0.003x + 0.1 cm, *r*^2^ = 0.002.

Bland–Altman plots displaying 95% limits of agreement for flight time and jump height (Figure 6) showed a high level of agreement since almost all paired measurements lie within ±1.96 * SD of the differences (dashed line). Figure 6 also shows low systematic bias ± random errors as 1.74 ± 14.19 ms and 0.18 ± 1.57 cm for flight time and jump height, respectively. Homoscedasticity of the errors was observed for both flight time and jump height (*r*^2^ = 0.003 and *r*^2^ = 0.002, respectively), indicating no association between the magnitude of the errors and the mean value [15,28].

**Figure 7:**
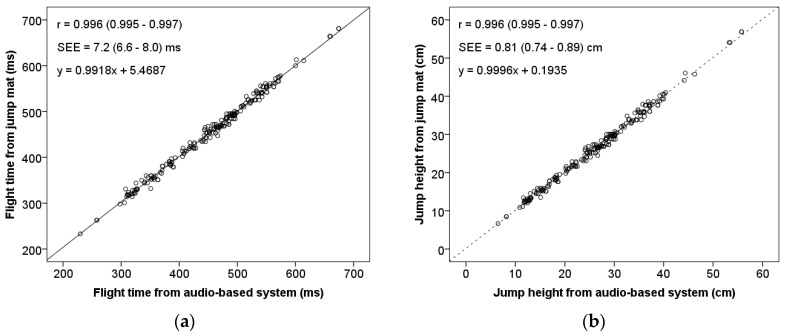
Relationship between measurements derived from jump mat system and audio-based system. (**a**) Flight time; (**b**) Jump height. Pearson’s product moment correlation coefficient (*r*) and standard error of estimate (SEE) shown with a 95% confidence interval between brackets; *p* < 0.01.

Pearson’s product moment correlation coefficient revealed almost perfect association between the audio-based system and jump (*r* = 0.996 for both measurement magnitudes, *p* < 0.01), as shown in Figure 7. The accuracy of the predictions made with the regression line is very high, as given by the SEE values of 7.2 (6.6–8.0) ms and 0.81 (0.74–0.89) cm of the flight time and jump height, respectively.

## 4. Discussion

The aim of this study was to develop a non-invasive instrument to measure jump height by automatically extracting take-off and landing events from audio recordings. Jump height has traditionally been measured with laboratory-based instruments, such as force plates [4] or 3D motion capture [6], or with simpler instruments through jump flight time, such as jump mats [9] or photocell mats [11]. While the latter have been very popular due to their portability and low cost, recently, smartphone apps aimed at measuring jump height have become widespread due to their portability, connectivity, and data processing abilities. However, the main drawback of such systems is the accuracy of measurements carried out through the manual digitization of video recordings with limited temporal resolution (fps) and time-consuming post processing.

The system developed in this study captures the sound wave pattern of jump executions to detect take-off and landing automatically, from which one can calculate jump height in real time without manual digitization at temporal resolutions that outperform those of flight-time-based mats (typically 1000 Hz) and high speed video rates from smartphones (typically 240 fps).

A tailored signal processing algorithm for audio event classification and detection has been developed for this application. Based on different spectral composition of take-off and landing phases in a jump execution, this algorithm is able to detect and classify such time events accurately. The time averaged energy on its correspondent frequency band has been used successfully as a simple threshold due to the high energy produced on landing. The take-off is detected inside a search time window before the landing. The different parameters of the algorithm have been optimized for minimum error by means of a sensitivity test using a training set of jumps. The algorithm has proven robust against different noise backgrounds and the reverberation characteristics of typical sports facilities where jump height tests are conducted.

A validation study was also carried out by comparing the measured jump heights from the audio-based system with those of a validated, commercial jump mat system [9]. There was almost perfect agreement between these instruments (ICC~1), indicating that the reliability of the audio-based system is practically perfect [15]. This statement is also supported by the narrow confidence intervals for flight time and jump height, consistency, and absolute agreement (0.995–0.997). Further, the agreement between the two measurements of the same variable is assessed by the currently recognized statistical method of Bland–Altman plots [23]. In this study, Bland–Altman plots demonstrated negligible systematic bias between instruments for jump height (0.18 cm). Therefore, the audio-based system can be regarded as a measurement tool with high accuracy. Similarly, the narrow limits of agreement (1.6 cm) suggest high precision of the measurement tool. Also, the plot shows that almost all the points are scattered on either side of the systematic bias within limits of agreement. The regression line and the Pearson’s product moment correlation coefficient of scattered data revealed no association between the magnitude of the errors and the mean value (*r*^2^ < 0.1) [24]. Such homoscedastic errors mean that, irrespective of the jump height score measured by the audio-based system, the amount of measurement errors will be stable. This feature is of paramount importance for assessing typical small changes in high-scoring athletes in response to an experimental intervention [28].

The minimum improvement in jump height likely to have a practical impact when assessing athletes is 1.9 cm, as a conservative fraction of the between-subjects SD [29]. The uncertainty of the measure is given by the standard error of estimate, which is very low for the audio-based system (0.8 cm or standardized 0.09, trivial effects), so the ratio between the smallest practical change for the athlete sample and the uncertainty (the so-called signal to noise ratio) is relatively large: 2.36 for the jump height. This means, in practice, that the developed audio-based system is a sensitive measurement tool to monitor changes in jump height scores over the potential uncertainty around the measure [22]. Our results also show that the audio-based system can provide valid measures of jump height, according to the almost perfect association between instruments (*r* = 0.996).

## 5. Conclusions

In this paper, we present a new instrument for measuring jump height through automatic extraction of flight time from audio signals captured in jump execution. To that end, a smartphone microphone is placed near athlete’s feet, whose shoe sole is attached with a piece of regular adhesive tape to facilitate identification of the take-off phase. A simple but effective audio signal processing algorithm accurately detects and discriminates the take-off and landing events, calculating the flight time with proven congruence, precision and noise immunity in real scenarios. Unlike video manual digitizing methods, the proposed technique developed in this paper is fast, non-invasive, simple to use, unattended, and lacks human observation errors. Further, the measurement methodology presented uses the capture audio system of a smartphone and Matlab code but can be readily automated as an app to facilitate its use both in laboratories and in the field. A multimodal system that uses sound and image at the same time could be implemented in the future in order to improve the system precision and immunity to false alarm events, such as impulsive noises that coincide in time with the jump execution.

## Figures and Tables

**Table 1:** Description of recording scenarios.

	Number of Jumps	Reverberation (T60)	Description
Gymnasium	80	1.3	High-volume room with plenty of people and sports equipment. Frequent impulsive noises of moderate intensity and constant background noise.
Workout room	75	0.7	Medium-sized room with moderate concurrence of people. Generally, background music is playing and occasional high-intensity impulse noises.
Noisy corridor	35	0.6	Longitudinal room with few people. High background noise from the computer hum of an adjacent room.
Small fitness room	35	0.35	Low-sized room with small groups of people. Low impulsive noises and moderate background noise, mainly from ambient music.

T60 (s) is an objective reverberation description, defined as the time it takes for sound to decay by 60 dB.

**Table 2:** Noise levels and signal-to-noise ratios of recording scenarios.

	Background Noise LP	Landing Signal Level	SNR Landing	Background Noise HP	Take-off Signal Level	SNR Take-off
Gymnasium	−29.3	−11.2	18.1	−37.4	−13.0	24.4
Workout room	−28.0	−10.5	17.5	−36.0	−13.3	22.7
Noisy corridor	−26.8	−8.3	18.5	−38.4	−15.2	23.2
Small fitness room	−37.4	−9.1	28.3	−35.7	−12.8	22.9

Background noise and signal level expressed in decibels full scale (dB_FS_), where 0 dB would correspond to the power of a square signal occupying 100% of dynamic range. Signal-to-noise ratio (SNR) expressed in decibels (dB). LP: Low Pass, HP: High Pass.

**Table 3:** Pairwise reliability of jump mat and audio-based system.

	Jump Mat vs. Audio-Based System	
	Flight Time	Jump Height
ICC (2,1)# (95% CI)	0.996 (0.995–0.997)	0.996 (0.995–0.997)
ICC (2,1)§ (95% CI)	0.996 (0.995–0.997)	0.996 (0.995–0.997)
Cronbach’s α	0.998	0.998
Mean difference (95% CI)	1.7 * (0.8–2.7) ms	0.18 * (0.08–0.29) cm
SWC (95% CI)	17.1 (15.6–18.9) ms	1.9 (1.7–2.1) cm
SEE (95% CI)	7.2 (6.62–8.01) ms	0.81 (0.74–0.89) cm
Standardized SEE (95% CI)	0.09 (0.07–0.10)	0.08 (0.07–0.10)
SEE Effect Size	Trivial	Trivial

Intra-class correlation coefficient (ICC) showing consistency (#) and absolute agreement (§) for the comparison between systems, 95% CI = 95% confidence interval; * *p* < 0.01.
